# Fate of Fertilizer Nitrogen in the Field 2 Years After Biochar Application

**DOI:** 10.3390/plants14050682

**Published:** 2025-02-23

**Authors:** Lining Zhao, Weijun Yang, Zi Wang, Jinshan Zhang, Liyue Zhang, Mei Yang, Xiangrui Meng, Lei Ma

**Affiliations:** College of Agronomy, Xinjiang Agricultural University, Urumqi 830052, China; zhaolining2222@163.com (L.Z.); wangzi11072023@163.com (Z.W.); zhangjinshan0530@sina.com (J.Z.); 15683879534@163.com (L.Z.); ymwql1995@163.com (M.Y.); mxr766618615@163.com (X.M.); mlei0526@163.com (L.M.)

**Keywords:** biochar, nitrogen fertilizer, ^15^N tracer technique

## Abstract

This study aimed to clarify the scientific quantification of fertilizer nitrogen (N) uptake and utilization, its destination, and its residual distribution in the soil at a depth of 0–30 cm after biochar application using ^15^N tracer technology. The purpose was to provide a theoretical basis for developing a scientific application strategy for N fertilizer and biochar in irrigated farmland areas. Two levels of N fertilizer application were set up using the ^15^N labeling method in microareas of large fields: the regular amount of N fertilizer (N1: 300 kg·ha^−1^) and a reduction of N fertilizer by 15% (N2: 255 kg·ha^−1^). Further, three levels of biochar application were set up: no biochar (B0: 0 kg·ha^−1^), a low amount of biochar (B1: 10 × 10^3^ kg·ha^−1^), and a medium amount of biochar (B2: 20 × 10^3^ kg·ha^−1^). The tested biochar was derived from corn stover (maize straw). The natural abundance of ^15^N-labeled fertilizer N, the total N content of each aboveground organ, and the total N content of soil at a depth of 0–30 cm in a spring wheat field at maturity were determined, and the yield was measured in the corresponding plots. The proportion of ^15^N-labeled fertilizer N uptake by each organ of spring wheat and the soil N uptake was 20.60–35.32% and more than 64.68%, respectively. Moreover, the proportion of soil N uptake showed a decreasing trend with an increase in biochar application. The spring wheat N uptake and utilization rate, the residue rate in the soil at a depth of 0–30 cm, the total utilization rate, and the rate of loss of ^15^N-labeled fertilizer N ranged from 15.21% to 29.61%, 23.33% to 28.93%, 38.54% to 58.54%, and 41.46% to 61.46%, respectively. The spring wheat N fertilizer utilization rate, fertilizer N residue rate in soil, and total fertilizer N utilization rate all increased gradually with an increase in biochar application, except for the N loss rate, which decreased gradually. When N fertilizer reduction was combined with medium biochar (B2N2), the yield of spring wheat significantly improved, mainly due to an increase in the number of grains in spikes. Under this treatment, the number of grains in spikes of spring wheat was 41.9, and the yield reached 7075.54 kg·ha^−1^, which was an increase of 9.69–28.25% and 10.91–25.35%, respectively, compared with other treatments. Yield increased by up to 25.35%, and nitrogen loss decreased by 48.24% under the B2N2 treatment. Biochar application could promote the amount and proportion of fertilizer N uptake in various organs of spring wheat as well as in the soil at a depth of 0–30 cm. In this study, a 15% reduction in N fertilizer (255 kg·ha^−1^) combined with 20 × 10^3^ kg·ha^−1^ biochar application initially helped achieve the goal of increasing spring wheat yield and N fertilizer uptake, as well as improving fertilizer N utilization, providing an optimal scientific application strategy for N fertilizer and biochar in the farmland of the irrigation area. These results substantiate the hypothesis that biochar application enhances spring wheat (*Triticum aestivum* L.) assimilation of fertilizer-derived nitrogen (^15^N) while concomitantly improving fertilizer nitrogen retention in the soil matrix, which could provide a sustainable framework for nitrogen management in irrigated farmlands.

## 1. Introduction

Over the past few decades, significant increases in agricultural production have been achieved at the expense of high resource input and severe environmental pollution. The excessive application of nitrogen (N) fertilizers has been identified as the primary factor contributing to the substantial decline in N use efficiency, which is only 30–50% [1]. Unused N fertilizers remain in the soil, escape into the atmosphere through ammonia volatilization and denitrification, and leach into deeper soil and groundwater. This leads to the waste of agricultural resources and affects the sustainable development of agricultural ecology. Biochar is a solid product formed by the pyrolysis of bio-organic materials in an anoxic or anaerobic environment [2]. It possesses several advantageous characteristics [3], such as a porous structure, a large specific surface area, strong adsorption capacity, and strong carbon stability. Therefore, it has attracted interest from researchers across multiple disciplines, including agriculture, the environment, and energy [4]. Recent studies have reported the active role played by biochar in improving the soil structure [5], hydraulic characteristics [6], soil fertility [7], control of nonpoint source pollution [8], and other aspects. Biochar can be used alone or as an additive to upgrade soil, improve fertilizer and water use efficiency, and reduce or avoid specific environmental pollution [9]. Despite extensive studies, the residual effects of biochar applied over two years on nitrogen dynamics in irrigated soils remain unclear.

The application of biochar significantly enhances the fixation, transformation, and utilization efficiency of nitrogen in the soil, thereby increasing crop yields while mitigating environmental impacts. Research has demonstrated that biochar treatment leads to a reduction in soil nitrate (N-NO_3_^−^) and dissolved organic carbon (DOC) levels, with the extent of reduction being dose-dependent [10]. A seven-year field experiment established six treatment groups, including a control group and various combinations of chemical fertilizers and biochar. The study found that long-term biochar application progressively increased corn yields, with the effect becoming more pronounced over time [11]. Another study on rice indicated that an appropriate amount of biochar not only increased plant height but also significantly enhanced dry matter accumulation in leaves, stems, and roots [12]. Biochar improves nitrogen fertilizer utilization efficiency through mechanisms such as nitrogen fixation and reduced nitrate leaching, thereby promoting yield increases in crops like wheat, sugar beet, and corn [13,14,15]. Overall, biochar effectively enhances nitrogen utilization efficiency in agricultural production. While current studies primarily evaluate effects under specific conditions (e.g., particular types of biochar or specific crops), future research should focus on cross-regional and multi-factor interaction studies to comprehensively understand biochar’s mechanisms.

The northern Xinjiang irrigation area is a paradigm of oasis irrigation agriculture, exhibiting robust grain production capacity in China. However, this region faces diminished soil organic matter, waning fertility, and erratic crop yields. The substantial yields of wheat, maize, and cotton frequently depend on the extensive application of fertilizers. Local farmers are generally accustomed to N input levels of 300 kg·ha^−1^, and the excessive application of N fertilizers has been demonstrated to reduce use efficiency, exacerbate greenhouse gas emissions, and create a series of environmental issues [16,17]. The long-term effects of biochar are remarkable in improving soil structure, enhancing soil fertility, and promoting carbon sequestration [18,19,20]. Therefore, understanding the loss pattern of crop-specific N fertilizers, the driving factors, and the mitigation potential is imperative for developing effective mitigation strategies. The ^15^N isotope tracer technique is a quantitative tool for determining the complex processes of N transformation within the soil–plant system. Studies have reported significant variations in the fate of N fertilizers and the resulting yield across different climates, soil properties, and field management practices [21]. Despite the extensive use of biochar in research aimed at enhancing soil fertility and increasing crop yield in irrigated oasis farmland, notable deficiencies have been observed in the existing research in the study region compared with research conducted in other regions. For instance, in the northern Xinjiang irrigation area, the mechanisms by which N fertilizer is absorbed by crops, remains in the soil, and is lost through various means following a single application of biochar and N fertilizer remain unclear. The addition of biochar increases N fertilization in cultivated wheat. Quantifying N loss pathways and analyzing the fate of N fertilizer are crucial for assessing the agronomic and environmental effects of N reduction combined with biochar application. This study aims to evaluate the effects of biochar on nitrogen retention, loss, and crop yield in irrigated farmland over two years.

Considering the present circumstances pertaining to the irrational application of N, such as in the irrigation area of northern Xinjiang, this study used ^15^N tracer technology to investigate the impact of varying amounts of N fertilizer in conjunction with biochar on the fate of N fertilizer in farmland. This study elucidated the absorption and utilization of ^15^N-labeled fertilizer N after the addition of biochar. The crop utilization, soil residue, and loss rate of fertilizer N in the farmland after biochar application were quantified. We hypothesize that biochar application under nitrogen fertilizer reduction regimes facilitates the achievement of “nitrogen use efficiency optimization” (NUE-O) objectives—characterized by maintaining grain yield parity while enhancing agronomic efficiency and ecological sustainability. This approach provides replicable solutions for resource-limited agricultural systems, demonstrating the potential to maximize the mitigation of environmental externalities (e.g., groundwater contamination) through enhanced nutrient retention and reduced reactive nitrogen losses. It could provide a theoretical basis for the rational application of fertilizer N and a scientific application strategy for biochar in the farmland of the irrigation area.

## 2. Results

### 2.1. N Accumulation in Aboveground Organs of Spring Wheat Plants at the Maturity Stage

**Figure 1:**
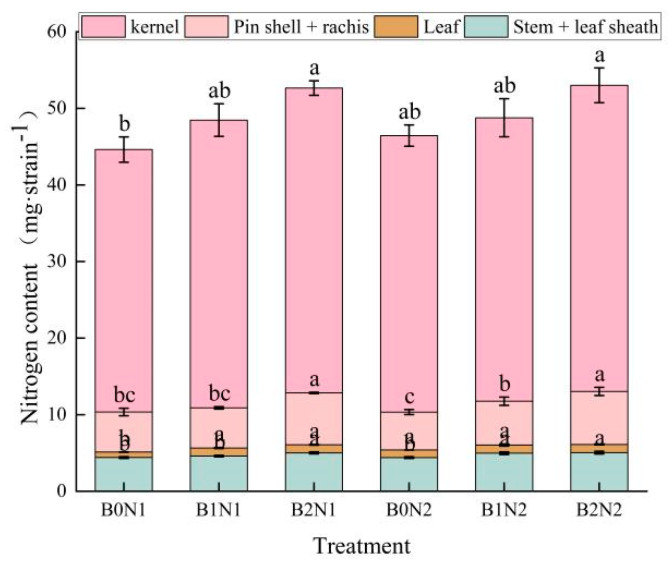
Nitrogen accumulation in aboveground organs of plants at maturity stage. Note: Different letters indicate significant differences (*p* < 0.05) among the treatments of biochar and nitrogen fertilizer application, while the same letters indicate no significant differences among the treatments.

As shown in Figure 1, biochar application and N fertilizer dosage optimization affected N accumulation in all organs of spring wheat at maturity. At the same N fertilizer dosage, N accumulation in each organ of spring wheat increased with the increase in biochar dosage. At the same dosage of biochar, reducing the dosage of N fertilizer increased N accumulation in the organs of spring wheat. The application of N fertilizer combined with biochar increased N accumulation by 0.59–14.55% in the stalks + leaf sheaths, 0.94–50.7% in the leaves, 2.36–40% in the pin shell + rachis, and 4–16.63% in the kernels of the spring wheat plant under the B2N2 treatment compared with other treatments.

### 2.2. Changes in ^15^N Abundance in Aboveground Organs of Plants at the Maturity Stage

**Figure 2:**
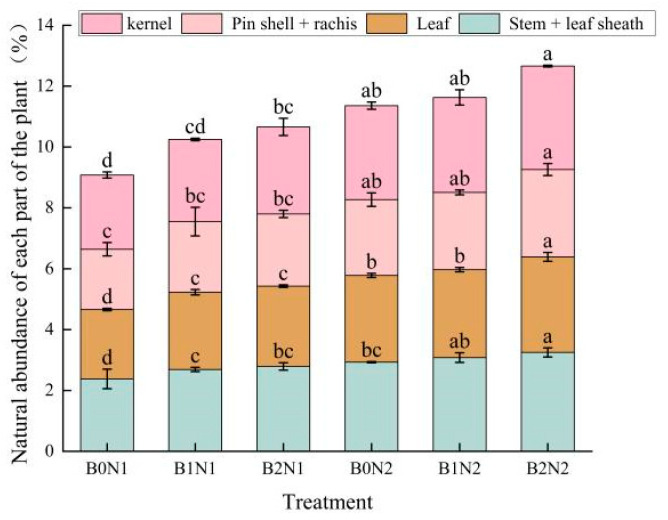
^15^N abundance in aboveground organs of spring wheat plants at maturity stage. Note: Different letters indicate significant differences (*p* < 0.05) among the treatments of biochar and nitrogen fertilizer application, while the same letters indicate no significant differences among the treatments, which is the same as below.

As shown in Figure 2, a significant difference (*p* < 0.05) was observed in the effect of different biochar application dosages and N fertilizer dosage optimization on ^15^N abundance in the aboveground organs of spring wheat at maturity. Under the same N application conditions, ^15^N abundance in the aboveground organs of spring wheat (except for the stems) increased with increasing biochar dosage. The values of ^15^N abundance in all organs of spring wheat were higher under the N fertilizer reduction treatment (N2) than under the conventional N application treatment (N1). The biochar application and N fertilizer reduction increased the ^15^N abundance values in the aboveground organs of spring wheat compared with the B0N1 treatment. The ^15^N abundance increased by 5.52–34.45% in the stalks + leaf sheaths, 11.40–37.72% in the leaves, 17.17–44.95% in the pin shell + rachis, and 10.66–39.34% in the kernels of spring wheat plants under the B2N2 treatment compared with other treatments.

### 2.3. Proportion of ^15^N-Labeled Fertilizer N and Soil N in Plants at the Maturity Stage

**Figure 3:**
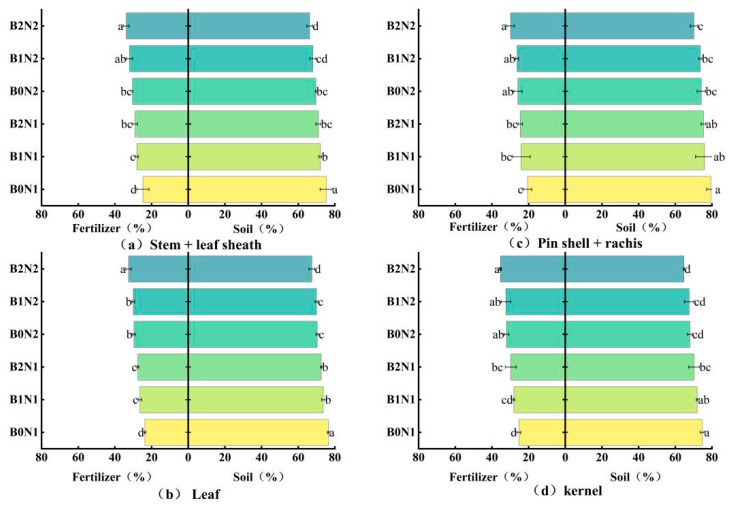
Absorption proportion of ^15^N-labeled fertilizer N and soil N in plant at maturity stage (%). Different N fertilizer sources for (**a**) stem + leaf sheath, (**b**) leaf, (**c**) pin shell + rachis, and (**d**) kernel. Different letters indicate significant differences (*p* < 0.05) among the treatments.

As shown in Figure 3, biochar application and N fertilizer dosage optimization significantly affected the uptake ratio of ^15^N-labeled fertilizer N in each organ of spring wheat plants and in soil N across different treatments. Under the same N application conditions, the percentage of ^15^N-labeled fertilizer N uptake by each organ increased, and the corresponding percentage of soil N uptake decreased with the increase in biochar application. At the same dosage of biochar, reducing the N fertilizer dosage prompted the plants to absorb more fertilizer N. Consequently, the percentage of ^15^N-labeled fertilizer N uptake by their organs increased. In contrast, the percentage of soil N uptake decreased accordingly. The combined effect of biochar and N fertilizer favored plant uptake of ^15^N fertilizer N. The percentage of ^15^N-labeled fertilizer N uptake by each aboveground organ of spring wheat was highest under the B2N2 treatment, reaching 29.82–35.32%.

### 2.4. Uptake and Utilization of ^15^N-Labeled Fertilizer N by Aboveground Organs of Spring Wheat at the Maturity Stage

**Figure 4:**
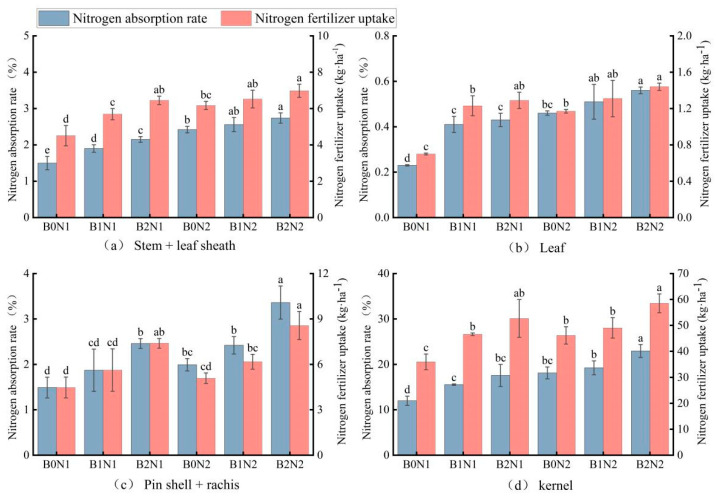
Absorption and utilization of ^15^N-labeled fertilizer N by aboveground organs of spring wheat. The fertilizer N uptake and utilization rate of (**a**) stem + leaf sheath, (**b**) of leaf, (**c**) pin shell + rachis, and (**d**) kernel. Different letters indicate significant differences (*p* < 0.05) among the treatments.

As shown in Figure 4, the effects of biochar application and optimized N fertilizer dosage on the uptake and utilization of N fertilizer by the organs of spring wheat were significantly different (*p* < 0.05). The trends in the uptake and utilization of N fertilizer by the organs of spring wheat under different charcoal and N rationing treatments were consistent. The highest uptake and utilization of ^15^N-labeled fertilizer N were observed in the seeds, at 35.95–58.51 kg·ha^−1^ and 11.98–22.94%, respectively. Under the same N application conditions, the uptake and utilization of ^15^N-labeled fertilizer N in all organs of spring wheat increased with the increase in biochar application. Under the B2N2 treatment, spring wheat organs showed the best performance in terms of fertilizer ^15^N uptake and utilization rate compared with other treatments. The N uptake increased by 7.06–54.77% in the stem, 9.92–105.71% in the leaf, 38.74–91.50% in the husk + spindle, and 19.31–62.75% in the seed.

### 2.5. ^15^N-Labeled Fertilizer N in Soil at a Depth of 0–30 cm in a Spring Wheat Field at the Maturity Stage

**Figure 5:**
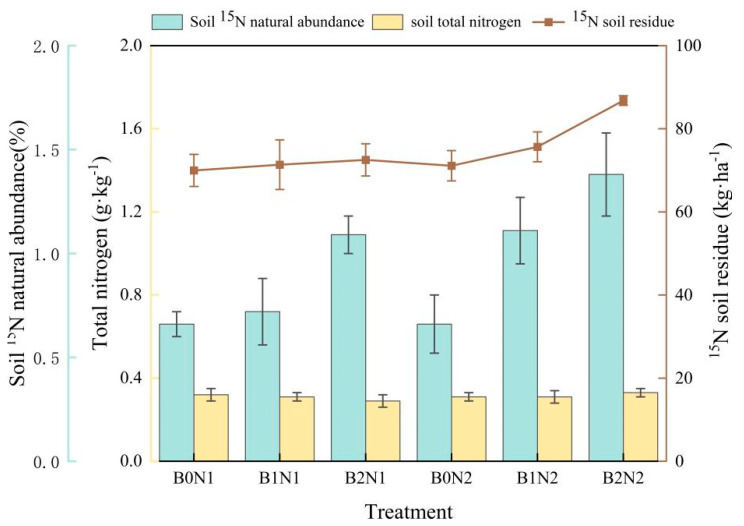
Effects of combined application of carbon and N on soil fertilizer ^15^N abundance, soil total N content, and soil residue amount.

The effects of biochar application and N fertilizer rate optimization on the natural abundance of ^15^N-labeled fertilizer N, soil total N content, and ^15^N soil residue were significantly different (*p* < 0.05). Biochar application promoted an increase in ^15^N abundance and its soil residue (Figure 5), which was even greater under reduced N fertilizer conditions. The highest ^15^N abundance and ^15^N soil residue were found under the B2N2 treatment, with respective rates of 1.38% and 86.80 kg·ha^−1^, compared with other treatments. Under reduced N fertilizer application (N2), the total N content in soil at a depth of 0–30 cm increased with the increase in biochar application. The highest soil total N content was 0.33 g·kg^−1^ under the B2N2 treatment, and the difference between treatments was not significant.

### 2.6. Fate of Fertilizer N

**Figure 6:**
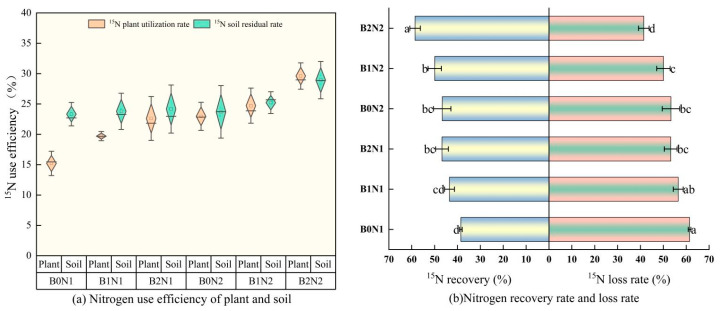
Fate of fertilizer N. (**a**) Utilization rate of ^15^N-labeled fertilizer N of plants and soil. (**b**) Total recovery rate and loss rate of ^15^N. Different letters indicate significant differences (*p* < 0.05) among the treatments.

As shown in Figure 6, the effects of biochar application and N fertilizer dosage optimization on ^15^N-labeled fertilizer N utilization, soil residue rate, total ^15^N uptake utilization, and ^15^N loss rate in spring wheat fields were significantly different among treatments (*p* < 0.05). The observed reduction in nitrogen loss correlates with increased soil retention capacity due to biochar. At the same N fertilizer dosage, increasing biochar promoted the utilization rate of ^15^N-labeled fertilizer N, soil residue rate, and ^15^N recovery rate in spring wheat plants, while subsequently reducing the ^15^N loss rate. The N fertilizer utilization rate of spring wheat plants under the B2N2 treatment increased by 19.78–94.67% compared with other treatments, the soil residue of fertilizer N increased by 14.71–24% compared with other charcoal and N dosing treatments, and the total ^15^N uptake and utilization rate increased by 14.67–51.89% compared with other charcoal and N dosing treatments, while the rate of ^15^N loss decreased by 20.72–48.24%.

### 2.7. Yield of Spring Wheat and Its Components

**Figure 7:**
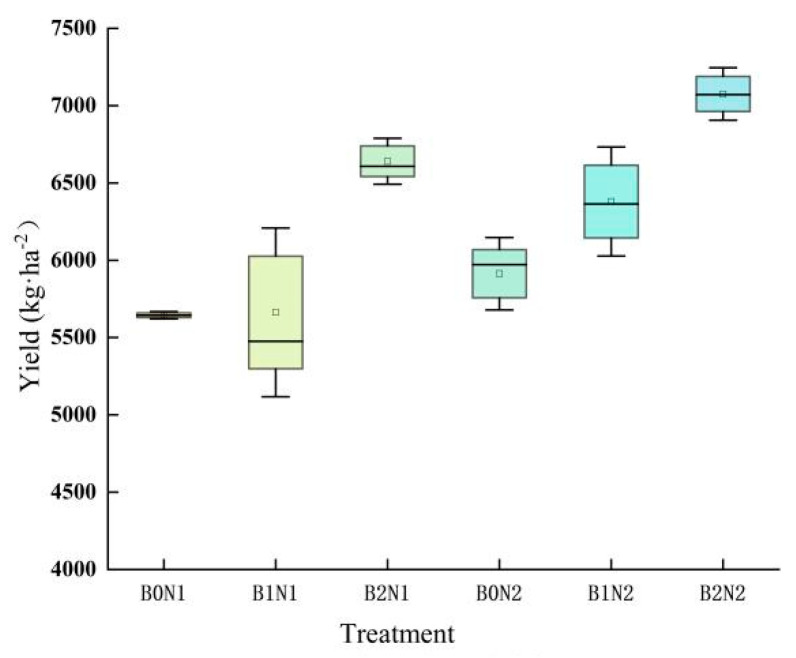
Spring wheat yield.

Biochar application and N fertilizer rate optimization affected the yield of spring wheat and its components (Figure 7). Under the same N application conditions, the number of grains, number of spikes, and yield of spring wheat showed an increasing trend with the increase in biochar application. Under the same charcoal application conditions, the number of grains in spikes, the number of spikes, and the yield of spring wheat all showed an increasing trend with the reduction in N fertilizer. Under the B2N2 treatment, the number of grains in spikes and the yield of spring wheat showed the best performance, at 41.90 and 7075.54 kg·ha^−1^, respectively. These values increased by 9.69–28.25% and 10.91–25.35%, respectively, compared with the other treatments.

**Figure 8:**
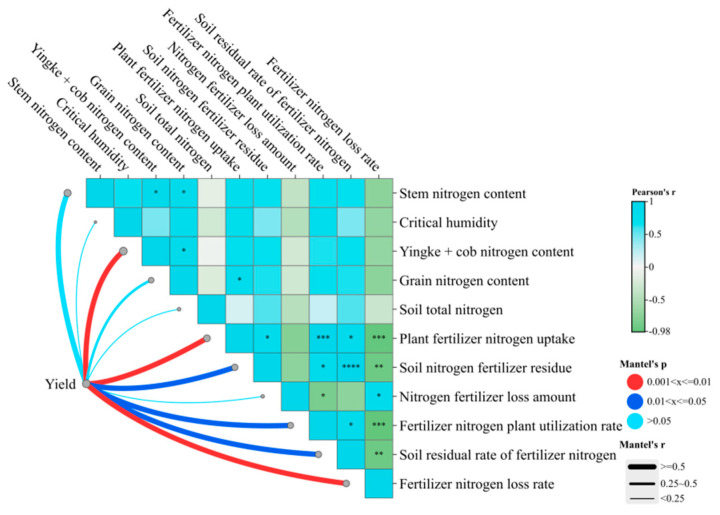
Analysis of the relationships between yield and N content of the plant, N absorption of plant fertilizer, and N loss rate. Different colors in the figure indicate whether the correlation between different indicators is significant, where “*” indicates a weak correlation; ** indicates moderately relevant. “***” indicates strong correlation; “****” is very relevant. The *p*-value is used to test whether the correlation is significant. The meaning is as follows: the red line is 0.001 < x ≤ 0.01, indicating a very significant correlation; The dark blue line indicates 0.01 < x ≤ 0.05, indicating a significant correlation; The sky blue line is x > 0.05, indicating no significant correlation.

The findings of the present study demonstrated significant relationships between the yield and the N content of Yingke + cob, N absorption of plant fertilizer, and N loss rate (0.001 < *p* ≤ 0.01) (Figure 8). Furthermore, the yield exhibited a significant correlation with soil N residue, plant utilization rate, and soil residue rate (0.01 < *p* ≤ 0.05). Conversely, the N loss rate negatively correlated with various indicators, except for N loss. This negative correlation was found to be statistically significant, particularly in relation to plant fertilizer N uptake, soil N residue, plant utilization rate, and soil residue rate.

## 3. Discussion

The ^15^N technique can be used to quantitatively indicate the fate of fertilizer N applied to agricultural land [22]. When ^15^N-labeled fertilizer N is applied to the soil, part of it is taken up by the crop, another part remains in the soil, and a small portion is lost through processes such as leaching and volatilization. Zhang et al. [23] conducted long-term ^15^N tracing and found that after more than 20 years of applying ^15^N fertilizers, only 61–65% of fertilizer N was taken up by the crop, and 12–15% of fertilizer N was still retained in the soil organic matter. Quan et al. [24] also investigated the fate of ^15^N fertilizer under different fertilizer application methods using a long-term experiment and found that fertilizer N fixed in microorganisms was significantly higher after the application of organic fertilizer during the early growth period of wheat. In this study, 2 years after a single application of biochar, 15.21–29.61% of fertilizer N was taken up by the crop, 23.33–28.93% remained in the soil, and 41.46–61.46% was lost. This can be attributed to the well-developed pore structure and large specific surface area of biochar, which can adsorb NH_4_^+^ and NO_3_^−^ in the soil, thus improving the soil’s N fixation capacity [25].

Kotuš et al. showed that biochar paired with N fertilizer affected N_2_O emissions [26]. Soils in the irrigation areas of the northern border were dominated by saline soils, whereas the higher pH of saline soils promoted ammonia volatilization. Further, the accumulation of inorganic N in the soil increased with the addition of biochar, which also increased the risk of soil NO_3_^−^-N loss [27]; biochar paired with N fertilizers increased the soil total N content [28]. The results of this study showed that the application of the conventional N fertilizer dosage in farmland caused greater losses. In contrast, reducing N fertilizer increased its use efficiency. When N fertilizer reduction was combined with a medium amount of biochar, ^15^N-labeled fertilizer N utilization significantly increased, and losses were reduced. This indicates that adding biochar, on one hand, optimized the microbial ecosystem of wheat field soil, enhanced the activity of soil N metabolism-related enzymes, and increased soil N content [25]. On the other hand, it improved the root vigor of spring wheat and promoted the root uptake and utilization of fertilizer N. The findings suggest that biochar can serve as a sustainable amendment, reducing nitrogen losses and improving agricultural productivity.

Biochar application could promote the uptake of ^15^N-labeled fertilizer N in spring wheat and reduce the N uptake from soil. Studies have shown that only 22.6–33.6% of the N absorbed by winter wheat comes from fertilizer N, and more than 66.0% relies on the uptake of soil N [29]. In this study, N fertilizer treatment alone showed a similar trend; however, when biochar was added with N fertilizer, the uptake capacity of ^15^N-labeled fertilizer N by wheat plants increased by 10.70–44.76%, and the N uptake from the soil decreased accordingly. When the application of N fertilizer was further reduced and biochar was applied, the N content, ^15^N abundance, and uptake of ^15^N-labeled fertilizer N in all aboveground organs of spring wheat increased, and the utilization rate also increased accordingly. This indicated, on one hand, that biochar influenced soil nitrification, which, in turn, affected the mutual conversion of NH_4_^+^-N and NO_3_^−^-N. This reduced the loss of N fertilizer through leaching and volatilization and enhanced N uptake and utilization by the crop [30]. On the other hand, the large pore space of biochar stored N fertilizer [31], delayed the release of adsorbed N, satisfied the sustained N supply for the growth and development processes of spring wheat, and promoted N uptake and utilization [32]. Some studies have shown that the total N content of soil increased significantly after 5 years of biochar application [33], and Zhou Pingyao et al. showed that the deep application of fertilizer significantly reduced N and phosphorus losses and improved the N fertilizer utilization rate in rice [34]. Further studies are required to validate these results across different soil types and climatic regions. In this study, reducing the amount of N fertilizer application and then pairing it with biochar significantly increased the total N content and ^15^N abundance in the soil at a depth of 0–30 cm. This can be attributed to the rich microporous structure and large specific surface area of the biochar itself [35], which adsorbed the fertilizer nutrients, reduced N loss, and improved the soil N supply capacity, thereby increasing the soil total N content and ^15^N abundance [36]. Also, the high C/N content of biochar provided a better living environment for soil microorganisms, promoted microbial activity, and effectively stimulated soil organic matter and total N content [37]. The positive excitation effect of biochar addition on soil also promoted soil nutrient operation [38], improved plant nutrient utilization [39], and thus promoted yield increase. This study was conducted in a controlled environment, and field-scale trials are necessary to confirm these results. Biochar enhances soil cation exchange capacity, improving nitrogen retention and reducing leaching. Therefore, reducing N fertilizer by 15% and combining it with biochar significantly increased the absorption and utilization of fertilizer ^15^N, as well as the soil residue rate, and significantly reduced the ^15^N loss rate of spring wheat. Both N fertilizer absorption and soil residue rate increased with the increase in the biochar application amount, promoting the absorption and utilization of N in wheat to improve the formation of seeds, which was conducive to yield improvement [40].

## 4. Materials and Methods

### 4.1. General Overview of the Test Site

A field positioning test was conducted at the wheat test station in Qitai County, Xinjiang (89°13′—91°22′ E, 42°45′—45°29′ N). The region experiences a temperate continental arid and semi-arid climate, which is characterized by an average annual temperature of 5.5 °C, a mean July temperature of 23.5 °C, an extreme maximum temperature reaching up to 42 °C, and an average annual precipitation of 269.4 mm. The soil is typical of the region, characterized by gray desert soil. The basic properties of the test site are presented in Table 1.

### 4.2. Test Materials

The test material, biochar, which was prepared from corn straw, was supplied by Liaoning Jinhefu Agricultural Science and Technology Co., Ltd. (Dandong, China). The fundamental properties of biochar are described in Table 2. The N fertilizer was ^15^N-labeled urea (Shanghai Research Institute of Chemical Industry, Shanghai, China; abundance of 10.12%). The variety of spring wheat used was “Xinchun 37”.

### 4.3. Experimental Design

The two-year timeline was chosen to investigate the residual effects of biochar. Based on a previous positioning experiment, this study established two N fertilizer levels in the main area: conventional N fertilizer (N1: 300 kg·ha^−1^) and N fertilizer reduced by 15% (N2: 255 kg·ha^−1^). In the sub-region, three biochar levels were implemented: no biochar (B0: 0 kg·ha^−1^), a low amount of biochar (B1: 10 × 10^3^ kg·ha^−1^), and a medium amount of biochar (B2: 20 × 10^3^ kg·ha^−1^). Biochar doses were selected based on previous studies indicating optimal soil improvements at these levels. The treatments without biochar were designated as B0N1, B1N1, and B2N1, whereas those with biochar were designated as B0N2, B1N2, and B2N2. Six treatments were implemented, with three replicates, each allocated to a plot area of 9 m^2^ (3 m × 3 m). The N fertilizer employed in the plot experiment was ordinary urea, with an N content of 46%. The N fertilization method employed was a basal and topdressing approach, with topdressing administered at the jointing stage and the booting stage in a ratio of 6:4. The irrigation method employed was drip irrigation, corresponding to the prevailing local management practices. The planting density of spring wheat was set at 450 × 10^4^ plants·ha^−1^, with a row spacing of 0.2 m.

**Figure 9:**
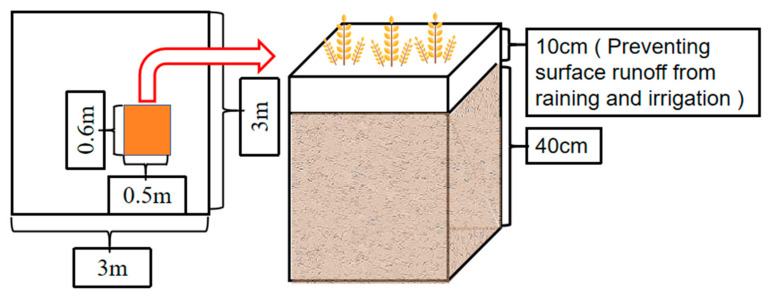
Microarea diagram.

A microarea of size 0.3 m^2^ was set up by placing bottomless galvanized iron with a length of 0.6 m, width of 0.5 m, and height of 0.5 m in the middle of the corresponding plot. The soil was plowed to a depth of 40 cm before sowing wheat in the spring of 2023, leaving 10 cm exposed on the ground surface to prevent the loss of the ^15^N-labeled N fertilizer during irrigation (Figure 9). The 0–20 cm topsoil in the microarea was removed, mixed with the ^15^N-labeled fertilizer, and backfilled into the microarea, the surface was flattened and compacted, and then the wheat was sown. Fertilizer was applied in a 5:5 base ratio, followed by a 6:4 ratio at the nodulation and spike stages, with no additional fertilizer applied thereafter. Biochar was evenly spread on the ground as a base fertilizer in April 2021. Then, the biochar was manually turned evenly into the tillage layer at a soil depth of 30 cm, with no further application at later stages. Soil and plant sampling for this experiment took place during the April–August 2023 spring wheat growing season.

### 4.4. Determination Items and Methods

#### 4.4.1. Collection of Plant and Soil Samples

Plant samples: Five wheat plants with uniform growth were randomly selected from the microarea at the maturity stage of spring wheat, sealed and stored in the laboratory, deactivated at 105 °C for 30 min, and dried to a constant weight.

Soil samples: After the maturity of spring wheat, soil samples from a depth of 0–30 cm were obtained using a five-point method from the microarea, the impurities were removed, and the samples were mixed evenly and brought back to the laboratory. The samples were air-dried at room temperature, passed through a 0.2 mm sieve, and then packaged.

Soil bulk density: The soil bulk density was measured using the ring knife method.

#### 4.4.2. Determination of Plant N and Soil N

The plant samples were divided into stems, leaves, cobs + glumes, and grains, and then ground through a 0.2 mm sieve. Plant N was determined using a fully automatic N analyzer after digestion with concentrated sulfuric acid. Soil total N was determined by digesting the soil samples and quantitatively transferring them to a semi-automatic Kjeldahl N analyzer after distillation using the semi-micro volume Kjeldahl method [41]. A 2 g air-dried soil sample was weighed and placed into a Kjeldahl flask. A mixture of concentrated sulfuric acid and potassium dichromate (K_2_Cr_2_O_7_) solution was added, followed by digestion via heating. After digestion, distilled water or tap water was added to the mixture and thoroughly mixed. The solution was then connected to a distillation apparatus, in which ammonia (NH_3_) was trapped using sodium hydroxide (NaOH). Finally, the liberated ammonia was titrated with a standardized hydrochloric acid (HCl) solution [42].

#### 4.4.3. Determination of ^15^N Abundance in Plant and Soil

The plant samples were divided into stems, leaves, rachis + glumes, and grains, and then separately ground and sieved through a 0.2 mm sieve. A 0.1 g aliquot of each sample was digested with 4 mL of concentrated sulfuric acid using a catalyst mixture of K_2_SO_4_:Se at a ratio of 500:1. After 8 h of digestion, the samples were absorbed using 0.02 mol L^−1^ dilute sulfuric acid and analyzed by isotope ratio mass spectrometry (Thermo-Fisher Delta V Advantage IRMS, Thermo Fisher Scientific, Waltham, MA, USA) to determine the ^15^N abundance in both plant tissues and soil.

#### 4.4.4. Determination of Spring Wheat Yield

At the mature stage of spring wheat, 1 m^2^ per plot was selected to determine the number of effective spikes of the wheat, and then 10 spring wheat plants with the same growth vigor were selected to test and calculate the theoretical yield.

#### 4.4.5. Soil Basic Physics and Chemistry

The organic matter content was quantified using the potassium dichromate oxidation-volumetric method. The total potassium (TK) content in the soil was analyzed through NaOH fusion followed by flame photometry. Total phosphorus was assessed via the NaOH molybdenum–antimony resistance colorimetric method. Soil alkali-hydrolyzable nitrogen was determined using the diffusion method after alkali hydrolysis. The available phosphorus was measured using sodium bicarbonate extraction coupled with the molybdenum–antimony resistance colorimetric method.

#### 4.4.6. Related Calculation

The plant ^15^N uptake (kg ha^−1^) and utilization ratio (%) in the microarea were calculated using the following equation [23]:

(1) Plant N15 uptake (kg ha−1)=Np(a−b)c−d

where *N_p_* is the total N uptake of wheat in the microarea (kg·ha^−1^), *a* is the ^15^N abundance detected in cotton plant samples (%), *b* is the natural abundance of ^15^N detected in the cotton plant (atom%, 0.3663%), *c* is the ^15^N enrichment of the labeled urea (atom%, 10.12%), and *d* is the natural abundance of ^15^N detected in fertilizer (atom%, 0.3663%).

(2) Plant N15 utilization ratio (%)=Plant N15 uptakeNF15

where ^15^N_F_ is the amount of isotope input.

The soil ^15^N uptake (kg·ha^−1^) and utilization ratio (%) in the microarea were calculated according to the following equation:

(3) Soil N15 uptake (kg ha−1)=e×d×TN×g×100

where *e* is the microarea soil thickness (30 cm), *d* is the microarea soil bulk density, TN is the soil total N in the microarea, and *g* is the percentage of ^15^N atoms in the microarea soil.

(4) Soil N15 utilization ratio=Soil N15 uptakeNF15

(5) N15 loss%=100−plant N15 utilization ratio−soil N15 utilization ratio

### 4.5. Statistical Analysis

Statistical analysis was performed using DPS V9.01 on all data (analysis of variance using the Duncan method). Data processing was performed using Excel 2019, and mapping was performed using Origin 2021. Differences were considered significant at *p* <0.05.

## 5. Conclusions

In conclusion, the addition of biochar could increase the uptake and proportion of ^15^N-labeled fertilizer N in the aboveground organs of spring wheat and increase the residual amount of ^15^N-labeled fertilizer N in the soil at a depth of 0–30 cm. A reduction of 15% in N fertilizer (255 kg·ha^−1^) and a biochar dose of 20 × 10^3^ kg·ha^−1^ (B2N2) could initially achieve the goals of reducing N fertilizer, improving fertilizer N utilization, and increasing the yield of spring wheat, with the yield reaching 7075.54 kg·ha^−1^. This study provides a theoretical basis for the rational optimization of N fertilizer dosage and the scientific application of biochar in irrigated farmland areas. Future research should focus on long-term nitrogen fertilizer labeling studies to elucidate the long-term impacts of varying biochar application rates in farmland, rather than overusing it, to fully leverage its potential.

## Figures and Tables

**Table 1:** Basic characteristics of the soil at the test site.

Year	Total Nitrogen (g·kg^−1^)	Total Phosphorus (g·kg^−1^)	Total Potassium (g·kg^−1^)	Organic Matter (g·kg^−1^)	Alkaline Hydrolysis of Nitrogen (mg·kg^−1^)	Quick-Acting Phosphorus (mg·kg^−1^)	pH
Winter wheat 2021–2022	1.09	1.22	28.21	18.40	69.32	17.96	8.34
Spring wheat in 2023	1.16	1.24	28.10	23.29	52.71	19.12	7.76

**Table 2:** Basic properties of biochar.

Biochar Material	Carbonization Temperature (°C)	Carbonization Time (h)	pH	Total Nitrogen (g·kg^−1^)	Total Phosphorus (g·kg^−1^)	Total Potassium (g·kg^−1^)	Alkaline Hydrolysis of Nitrogen (mg·kg^−1^)	Quick-Acting Phosphorus (mg·kg^−1^)
Corn stalk biochar	450	4	9.3	21.7	10.5	21.4	5.3	200.9

## Data Availability

The datasets generated and/or analyzed during this study are available from the corresponding author upon reasonable request.
